# Ten-m3 Is Required for the Development of Topography in the Ipsilateral Retinocollicular Pathway

**DOI:** 10.1371/journal.pone.0043083

**Published:** 2012-09-19

**Authors:** Nuwan Dharmaratne, Kelly A. Glendining, Timothy R. Young, Heidi Tran, Atomu Sawatari, Catherine A. Leamey

**Affiliations:** Discipline of Physiology, School of Medical Sciences and Bosch Institute, University of Sydney, New South Wales, Australia; Tokyo Medical and Dental University, Japan

## Abstract

**Background:**

The alignment of ipsilaterally and contralaterally projecting retinal axons that view the same part of visual space is fundamental to binocular vision. While much progress has been made regarding the mechanisms which regulate contralateral topography, very little is known of the mechanisms which regulate the mapping of ipsilateral axons such that they align with their contralateral counterparts.

**Results:**

Using the advantageous model provided by the mouse retinocollicular pathway, we have performed anterograde tracing experiments which demonstrate that ipsilateral retinal axons begin to form terminal zones (TZs) in the superior colliculus (SC), within the first few postnatal days. These appear mature by postnatal day 11. Importantly, TZs formed by ipsilaterally-projecting retinal axons are spatially offset from those of contralaterally-projecting axons arising from the same retinotopic location from the outset. This pattern is consistent with that required for adult visuotopy. We further demonstrate that a member of the Ten-m/Odz/Teneurin family of homophilic transmembrane glycoproteins, Ten-m3, is an essential regulator of ipsilateral retinocollicular topography. *Ten-m3* mRNA is expressed in a high-medial to low-lateral gradient in the developing SC. This corresponds topographically with its high-ventral to low-dorsal retinal gradient. In Ten-m3 knockout mice, contralateral ventrotemporal axons appropriately target rostromedial SC, whereas ipsilateral axons exhibit dramatic targeting errors along both the mediolateral and rostrocaudal axes of the SC, with a caudal shift of the primary TZ, as well as the formation of secondary, caudolaterally displaced TZs. In addition to these dramatic ipsilateral-specific mapping errors, both contralateral and ipsilateral retinocollicular TZs exhibit more subtle changes in morphology.

**Conclusions:**

We conclude that important aspects of adult visuotopy are established via the differential sensitivity of ipsilateral and contralateral axons to intrinsic guidance cues. Further, we show that Ten-m3 plays a critical role in this process and is particularly important for the mapping of the ipsilateral retinocollicular pathway.

## Background

The superior colliculus (SC) is a major retinal target in rodents. Its highly stereotyped topography can be largely described in two-dimensions, making it an excellent model for unravelling visuotopic mapping mechanisms. There is compelling evidence that axonal guidance molecules play important roles in retinal mapping within this structure.

In mice, contralaterally-projecting retinal ganglion cell (RGC) axons arise from the entire retina [1]. They initially overshoot their target zones in the SC and form retinotopically appropriate connections via interstitial branching [2]. Interactions between the EphA family of receptor tyrosine kinases and their ligands, the ephrinAs, help to mediate mapping of the temporonasal retinal axis onto the rostrocaudal (RC) collicular axis [3], [4], [5], [6]. EphB-ephrinB [2], [7], bone-morphogenic protein 4 (BMP4) [8] and Wnt-Ryk interactions [9] are implicated in mapping the ventrodorsal retinal axis onto the mediolateral (ML) SC axis.

**Figure 1:**
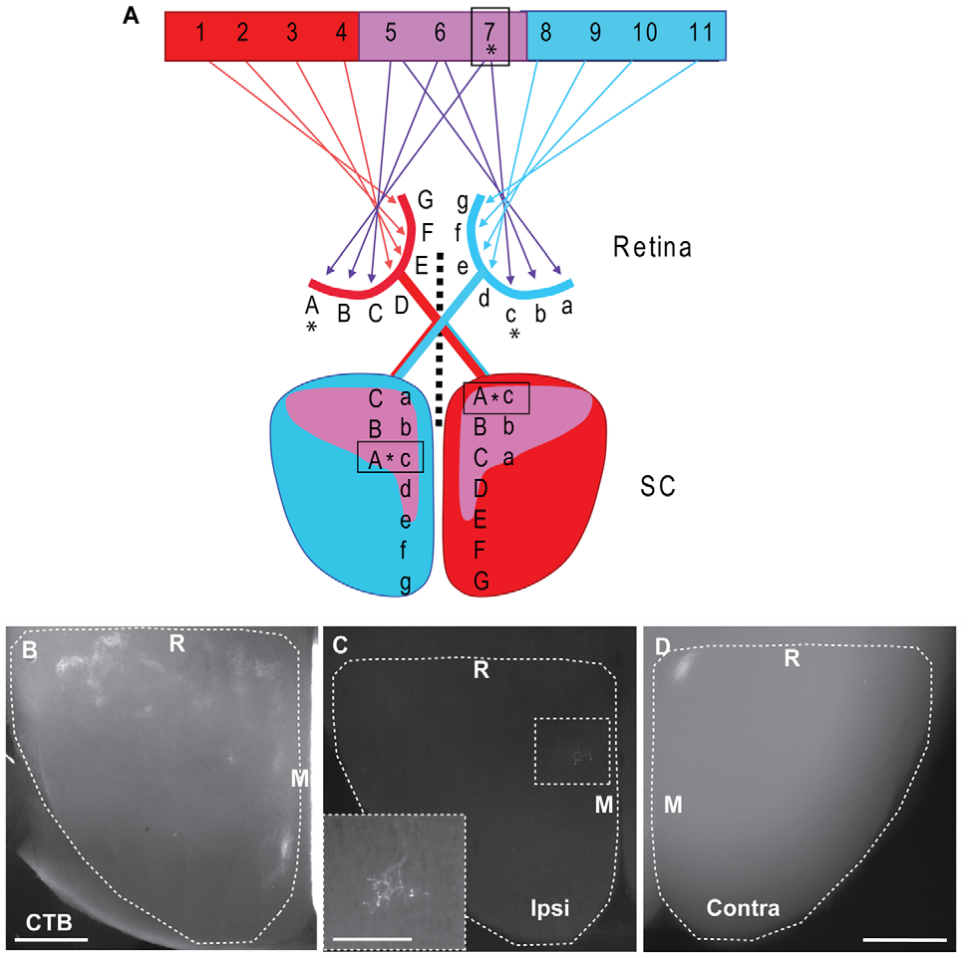
Schematic diagram and DiI tracing illustrating the visuotopic organisation of the retinocollicular pathway from ventrotemporal retina in the mature mouse. **A:** Left eye (red) G-A, representing visual field 1–7 projects topographically to the contralateral SC (red). Similarly, the right eye (blue) a–g, represents visual field 5–11 which projects topographically to the left SC (blue). The binocular field (centre, purple) 5–7 is represented by the ventrotemporal retina of both eyes (C-A, red & a–c, blue) and is the origin of the ipsilateral projection. The ipsilateral projection maps in reverse retinotopic order to produce an aligned map in the SC. Note that a given point in the visual field (e.g. 7,*) falls on different locations in the two retinas (red A & blue c, *). In order to generate an aligned map, these different retinotopic locations must project to the same region in the target (boxed regions, *). Conversely, the same retinotopic locations (red A & blue a) map to two different locations in the SC. When considering the ipsilateral and contralateral projections arising from a given retinotopic location from a single eye (e.g. red A, *), these will project to distinct regions in the contralateral and ipsilateral SC. Note the differing positions of the representation of red A in each collicular hemisphere (boxes, *). **B:** Horizontal section, approximately 300 µm below the surface of a flattened SC, from a P28 mouse following an injection of CTB into the ipsilateral eye to label all RGC axons. Ipsilateral terminals are arranged in clusters in the rostromedial region of the SC. The majority of the ipsilateral projection is visible in this single section. **C–D:** Horizontal section through the ipsilateral (ipsi) SC (C) and a whole-mount preparation of the contralateral (contra) SC (D) following a focal injection of DiI into the VTC from a P28 mouse. A dense contralateral TZ is visible at the rostromedial corner of the SC in D. In the ipsilateral SC (C) the TZ is offset caudolaterally from the contralateral TZ. The ipsilateral TZ is much less dense than the corresponding contralateral TZ and is shown at higher power in the inset. Scale bars: 500 µm. Scale in D also applies to C. Scale in inset in B: 200 µm. R: Rostral, M: Medial. Dashed lines delineate the borders of the SC.

In binocular species, retinotopic mapping alone is not sufficient to generate an accurate representation of visual space. To form a visuotopically-aligned map, the representation of the ipsilateral eye must invert across the temporonasal retinal axis with respect to contralateral projections (Fig. 1A; [10]). In mice, ipsilaterally-projecting RGCs arise from the ventrotemporal crescent (VTC) [1]. They initially project widely across the SC, and subsequently refine to occupy rostromedial regions [11], [12]. Refinement continues past eye-opening, suggesting a role for visual experience [12], [13]. These observations do not, however, address the generation of visuotopy. EphA-ephrinA interactions are involved in ipsilateral mapping, as deletion of ephrinA ligands disrupts the targeting of ipsilateral retinocollicular [14] and retinogeniculate [15] projections; contralateral mapping is similarly affected by these mutations. The generation of visuotopy requires ipsilateral and contralateral axons from the same retinotopic location to map differently in their targets (Fig. 1), suggesting that other cues may also be involved.

Ten-m3 is a member of the Ten-m/Odz/Teneurin family of homophilic transmembrane glycoproteins [16], [17]. Ten-ms are implicated in the regulation of many aspects of neural connectivity [18], [19], [20], [21], [22], [23], [24]. Recently, they have been shown to play critical roles in axon pathfinding [25], as well as synaptic organisation and targeting in the olfactory [26] and motor systems [27] in invertebrates. We initially identified Ten-m3 as a potential regulator of visual connectivity in mammals [23]. It is expressed in topographically corresponding gradients in retina, thalamus and cortex and regulates the targeting of ipsilateral inputs to the geniculocortical pathway. Deletion causes profound visual deficits [23], [28]. Its role in SC mapping, however, has not been described.

The complex, three-dimensional nature of topography within the dorsal lateral geniculate nucleus (dLGN) makes it difficult to determine whether the changes previously observed in retinogeniculate projections in Ten-m3 knockout (KO) mice [23] predominantly reflect changes along the representation of the temporonasal or dorsoventral retinal axes, or a combination of the two. Further, the organisation of the dLGN also makes it possible to overlook subtle mapping changes that may be critical for understanding the role of Ten-m3 and its potential mechanisms of action.

We here describe the role of Ten-m3 in retinocollicular development. As the normal development of ipsilateral retinocollicular topography has not been previously described, this was also investigated. We show that ipsilateral axons form topographically appropriate arbours via interstitial branching which commences within the first few postnatal days. Importantly, ipsilateral terminals are always offset, in a visuotopically appropriate manner, from corresponding contralateral terminals. We further reveal that Ten-m3 is expressed in a decreasing gradient across the ML axis of the SC which corresponds topographically to its decreasing ventrodorsal retinal gradient. Most significantly, we demonstrate that Ten-m3 plays a critical role in the targeting of ipsilateral retinocollicular axons. In its absence, ipsilateral retinocollicular projections make dramatic mapping errors. In addition, both ipsilateral and contralateral axons also show subtle changes affecting the shape of their TZs. Our data demonstrate that visuotopy arises via the differential response of ipsilateral and contralateral retinal axons to guidance cues, and that Ten-m3 is critical for the correct targeting of ipsilateral retinocollicular projections.

## Methods

### Ethics Statement

All experiments were conducted on mice. Protocols were approved by the University of Sydney Animal Ethics Committee (protocols 4634 and 5424), in accordance with National Health and Medical Research Council (Australia) guidelines. All recovery surgery was performed under isofluorane anaesthesia and euthanasia was performed with an overdose of sodium pentobarbital. All efforts were made to minimise suffering.

### Animals

Developmental experiments were conducted on C57/Black6 mice. Homozygous *Ten-m3* ^−/−^ (knockout, KO) and *Ten-m3* ^+/+^ (wild type, WT) mice were obtained by breeding pairs of *Ten-m3* ^+/−^ heterozygotes maintained on a C57/Black6× Sv129 background, as described previously [23]. The mixed background is required for the survival of Ten-m3 KOs. This produces animals of mixed pigment. Since albinism is associated with alterations in the ipsilateral retinal pathway, only results obtained from animals with highly pigmented retinas are included here. Genotyping was performed by polymerase chain reaction (PCR) using DNA isolated from tail biopsies.

### Tracer injections

All recovery surgeries were performed under anaesthesia induced and maintained by inhalation of 2–4% isofluorane in oxygen. The overall distribution of ipsilateral axons was assessed by bulk-fill injections of cholera toxin subunit B (CTB) conjugated with Alexa Fluor 488 (green) or 598 (red) tags (Invitrogen). For the study of normal development this was performed in 2 animals at postnatal day (P)0, and in 3 cases at P26. For analysis of the Ten-m3 phenotype, injections were performed at P14 in 8 KO and 7 WT mice. An injection of 0.5–1 µL of 1% CTB was made into the vitreous chamber of the eye in anaesthetised animals and 24–48 hours was allowed for dye transport. Animals were euthanised with an overdose of sodium pentobarbital (<100 mg/kg, i.p.) and intracardially perfused with 0.9% saline followed by 4% paraformaldehyde solution dissolved in 0.1 M phosphate buffer (pH 7.4).

The mature topography and normal development of the retinocollicular projection was investigated using focal injections of the lipophilic dye dioctadecyl-3-3-3′-3′-tetramethyl-indocarbocyanine perchlorate (DiI; Molecular Probes) at six ages: P0–1, P3–4, P7–8, P10–11, P16–18 and P26–28. Results reported are based on 2–5 animals for each age group. To address the role of Ten-m3 in the generation of retinal maps, focal tracer injections were also performed in Ten-m3 KO and WT littermates at P10. Results reported are based on 6 Ten-m3 KO and 4 WT littermates. It should be noted that the numbers quoted above are those in which good ipsilateral labelling was achieved and thus were included in the analyses. In a number of cases spanning both genotypes (around half of the total injections performed), although clear labelling of the contralateral projection was achieved, no label was observed in the ipsilateral SC, even though ipsilateral axons were observed to terminate in the pretectum in some of these cases.

Focal injections of DiI were made into the VTC of the retina in anesthetised mice. In animals with unopened eyelids (P10 and younger), the palpebral fissure was cut to allow access to the eye. The retina was then exposed by making a small hole in the sclera. A fine glass micropipette connected to a Pico pump pressure system was used to deliver a small volume (approximately 0.01–0.02 µL) of 10% DiI in dimethyl formamide (Sigma) into the retina. For animals younger than P11, the eyelid was sutured closed post-surgery. Mice survived for 16–48 hours to allow adequate dye transport before being euthanised and perfused as above.

SCs were dissected, flattened between glass slides and postfixed in 4% paraformaldehyde in 0.1 M PB before being viewed and photographed as whole mounts using epifluorescence optics on a Zeiss Axioplan 2 deconvolution microscope attached to a Zeiss AxioCam HR digital monochrome camera. This method was sufficient to clearly visualise axons in the ipsilateral SC of animals injected on P0 and P3 only. In older animals, the ipsilateral label was either blurred or completely obscured, even though contralateral label was apparent. In order to clearly visualise the ipsilateral label in animals from P7 and older, the SCs were embedded in agar or a solution of gelatine and albumin hardened with glutaraldehyde, and sectioned horizontally at 150 µm using a vibratome before being viewed and photographed as above. In some animals injected with CTB, 60 µm thick coronal sections through the SC were prepared using a freezing microtome. SCs were not flattened prior to coronal sectioning.

For the retinas, a cut was made in the sclera of either dorsal or nasal retina to maintain orientation. The eye was removed from the head and a cut was then made from the orientation marker towards the optic disc to act as a permanent fiducial. Retinas were dissected out, whole-mounted, viewed and photographed as above. These were used to determine the accuracy and document the location of the injection sites. Any cases with injections not well-localised to the appropriate region were discarded.

### Analysis

Images of the SC and retina were compiled into montages using Adobe Photoshop. Areas of TZs and the SC were measured from the photomontages using Image J (NIH). The ML and RC dimensions of the TZs and SC were similarly determined. Absolute measurements of TZs were normalised to control for any potential changes in SC size or shape by dividing by the appropriate measurement (area, RC or ML axis) for the entire SC and converting to a percentage. The RC:ML ratio of TZs was defined as the % of the RC SC axis encompassed by the RC axis of the TZ divided by the % ML SC axis encompassed by the ML axis of the TZ. In order to analyse CTB labelling, images of horizontal sections were thresholded using Image J. Image sizes were normalised and a heat-map was generated using a custom Matlab (Mathworks) program. Differences between overall WT and KO patterns were determined by subtracting WT from KO heat-map values. Statistical analyses were performed in Excel and SPSS using Student's t-test, ANOVA and, where appropriate, post-hoc tests.

### Ten-m3 expression studies

Analysis of Ten-m3 expression patterns was performed using *in situ* hybridisation and reverse-transcription, real-time, quantitative, PCR (qPCR). Embryonic day (E)16, P0 and P3 C57/Black6 mice were euthanised as above and decapitated. The retina was divided into dorsal, ventral, nasal and temporal quadrants. The SC was dissected into medial and lateral halves and tissue placed in RNAlater (Ambion). Total RNA was extracted, cDNA synthesised, and used to perform real-time qPCR as described in [19]. *In situ* hybridisation was performed on 15 µm thick fresh-frozen cryostat sections using digoxigenin labelled probes as described in [19]. Embryonic material was obtained from timed pregnancies. Dams were euthanised with an overdose of sodium pentobarbital and embryos were removed, decapitated and the heads frozen in isopentane on dry ice. Postnatal mice were euthanised as above before being decapitated. Brains were removed and frozen as above.

## Results

### Mature Organisation

A number of studies have described the overall projection pattern of the ipsilateral retinocollicular pathway in rodents. Ipsilateral axons are consistently reported to form clusters in the deep part of the *stratum griseum superficiale* (SGS) and the *stratum opticum* (SO) within the rostromedial SC [11], [29]. The topography of the ipsilateral retinocollicular pathway of the mature mouse has, however, not previously been characterised using anatomical approaches. We therefore examined this as a prelude to the developmental studies using horizontal sections through the flattened SC in order to gain maximum information regarding the spatial relationship between ipsilateral and contralateral projections when the projection is mature, at P28 [12]. The effectiveness of this procedure was initially confirmed following bulk-fill injections of CTB conjugated to fluorescent tags at P26 which were analysed at P28. This revealed clusters of ipsilateral terminals restricted to the rostral and medial SC, 300–400 µm deep to the pial surface (Fig. 1B). The use of fairly thick (150 µm) sections meant the bulk of the ipsilateral label was contained within a single section, greatly simplifying subsequent analysis.

Focal injections of DiI into the ventrotemporal retina consistently produced a contralateral TZ at the rostromedial border of the SC (Fig. 1D) and an ipsilateral TZ (Fig. 1C) which was offset both caudally and laterally from the contralateral TZ in the opposite hemisphere. Injections into more temporal or more ventral regions of the VTC produced the expected topographic shift for both ipsilateral and contralateral projections (Fig. S1). The RC offset is consistent with what would be required to generate visuotopy (Fig. 1A). Although dorsoventral mapping does not require a reversal of the ipsilateral projection in order to attain visuotopic alignment, a medial offset is predicted by the regions of the two eyes which are in binocular correspondence [1].

### Developmental studies

In order to characterise retinocollicular projections at the earliest possible postnatal age *in vivo*, animals were injected with DiI at P0 and examined at P1. Qualitative results are based on six animals; four with focal DiI injections into the peripheral ventrotemporal retina and two with intraocular injections of CTB to label all RGCs.

**Figure 2:**
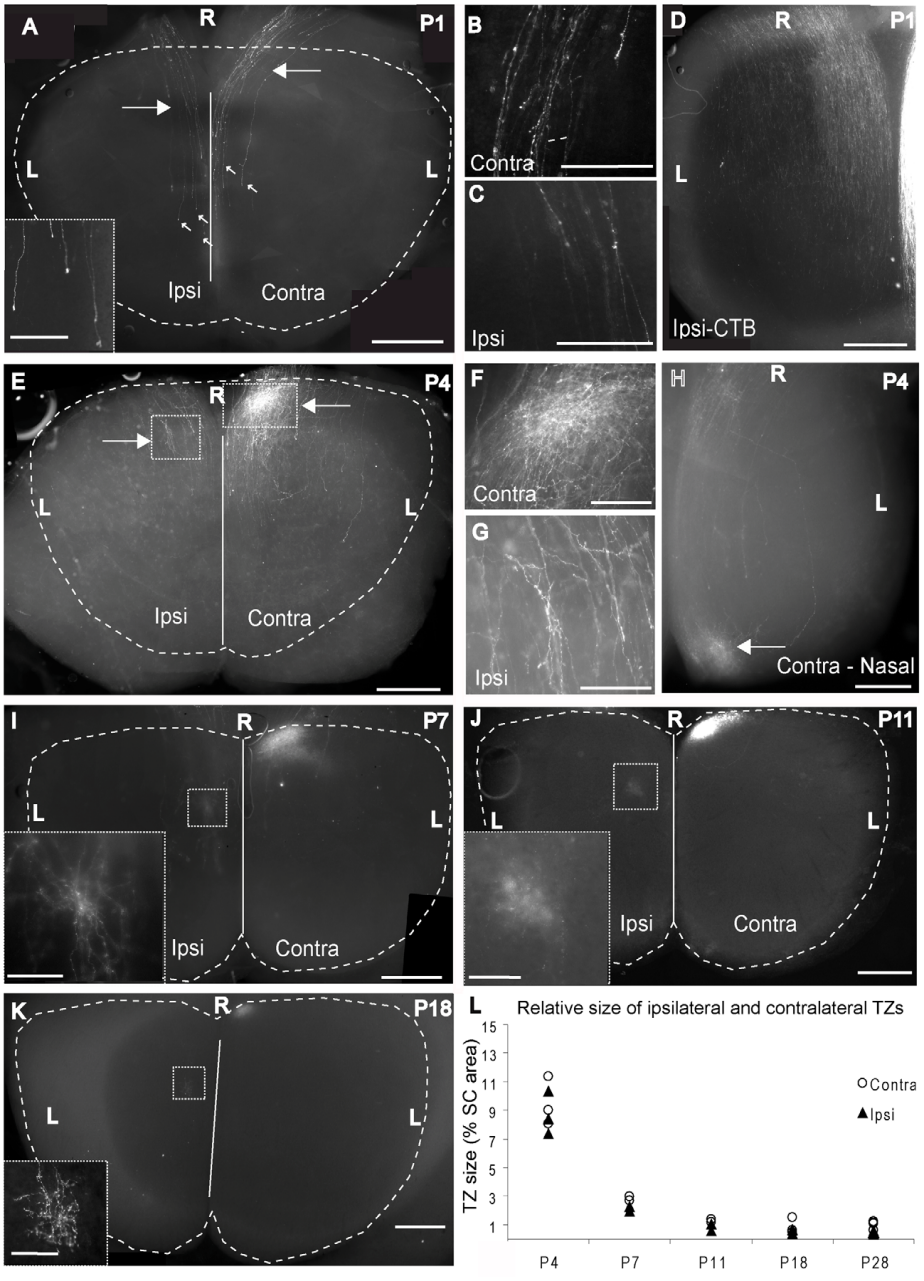
The ipsilateral TZ forms in the visuotopically appropriate region from early stages of development. **A–D:** The retinocollicular projection at P1 visualised in whole-mount preparations following injections of DiI (A–C) and CTB (D). Focal injection of DiI into the VTC at P0 reveals retinal axons coursing throughout much of the RC extent of the ipsilateral (ipsi) and contralateral (contra) SC (A). Axons are largely unbranched, including within the prospective TZ regions for both ipsilateral and contralateral axons. These regions are highlighted with arrows and are shown at higher power in B (contra) and C (ipsi). Axons continue caudally past this region and are tipped with growth cones (small arrows highlight growth cones, ipsilateral growth cones are shown at higher power in the Inset in A). Ipsilateral and contralateral axons show a bias towards medial SC. The overall distribution of ipsilateral axons labelled with CTB is visible in D. Axons predominantly target medial SC. **E–H:** The retinocollicular projection at P4 visualised in whole-mount preparations following injections of DiI into the VTC (E–G) or contralateral nasal (Contra - Nas) retina (H). A TZ, characterised by an increase in the density of label is beginning to emerge in contralateral SC (boxed area in E, right side, shown at higher power in F). The ipsilateral TZ (boxed area in E, left side) is less obvious but an area of increased labelling density is apparent; this is shown at higher power in G. The labelling pattern produced by an equivalent injection into nasal retina is shown for comparison in H, characterised by a clear TZ in contralateral caudal SC. No ipsilateral label was associated with this injection (not shown). **I:** Wholemount of SC at P7 following an injection of DiI into the VTC. The contralateral TZ is denser and better circumscribed than at P4, although some axons continue caudally past the TZ. The ipsilateral TZ (boxed area) appears diffuse in this preparation, indicating it is located deep to the surface. It can be seen more clearly in the inset which is taken from a horizontal section through the ipsilateral TZ. While it is more clearly defined than at P4, a number of axons continue caudally for some distance. The black box in the bottom right of the image corresponds to a small, unlabelled region of the SC that was inadvertently missed during the photomontage process. **J:** Horizontal section through the SC at P11 following a DiI injection into VTC. The contralateral and ipsilateral (boxed area, shown at higher power in the inset) TZs are now well circumscribed. **K:** As for J, but from a P18 mouse. Projections show little change from P11. **L:** Graph plotting the size of the ipsilateral and contralateral TZs relative to SC area across development. Symbols represent individual cases. The size of both decreases significantly between P4 and P7 (p<0.001, see results) and further between P7 and P11 (p<0.05, see results). No significant change is observed subsequent to P11 (p>0.9). Scale bars: A, D, E, H, I, J, K: 500 µm; B, C, F, G: 250 µm; insets in A, I, J, K: 100 µm. R: Rostral; L: Lateral. Dashed lines delineate the borders of the SC. Solid lines mark the midline.

By P1, DiI labelled fibres tipped with growth cones were seen coursing along the RC collicular axis on both sides of the midline (Fig. 2A). The fibres were largely unbranched, including within the regions of the presumptive future TZs for both contralateral (Fig. 2B) and ipsilateral axons (Fig. 2C). The lack of branching observed suggests that RGC axons from peripheral VT retina enter the SC on, or shortly before, the day of birth, consistent with previous studies [11]. Both ipsilateral and contralateral axons showed a bias towards the medial half of the SC, consistent with the adult topography. The number of labelled axons on the contralateral side was slightly higher than on the ipsilateral side. Ipsilateral axons consistently reached slightly more caudal levels compared to their contralateral counterparts. The clarity and high intensity of DiI label in SC whole mounts also suggests that both contralateral and ipsilateral axons are fairly superficial at this time point. Bulk-fill injections confirmed the bias of the ipsilateral projection to the medial half of the SC (Fig. 2D). In contrast to the placement of axons along the ML axis, the distribution of axons along the RC axis did not accurately reflect adult topography (Fig. 2A) at this stage of development. In all cases studied, axons were observed heading towards the caudal border, where they terminated in growth cones (Fig. 2A, inset), well past the location of the presumptive TZ in rostromedial SC.

By P4, axons had begun to ramify more extensively, and presumptive TZs could be identified. Comparison with previous studies indicates that these form predominantly in the deep SGS and SO from the outset [11]. Following injections into VT retina the contralateral TZ was apparent near the rostromedial border of the SC (Fig. 2E, F). Some axons continued caudally past the main part of the TZ, but the caudal overshoot was markedly less than at P0. The ipsilateral TZ was less obvious than the contralateral TZ (Fig. 2E), consistent with the much smaller ipsilateral projection in the adult [1]. The difference in the relative numbers of labelled ipsilateral and contralateral axons was more marked than at P1. This change may be due to cell death which peaks at P2 for the RGC population in the mouse [30]. At higher power (Fig. 2G), ipsilateral axons were seen to extend branches in a localized region which was offset both caudally and laterally from the contralateral TZ. As observed for the contralateral projection, axons overshot the TZ region caudally, but to a lesser extent than at P0. The difference in the position of the terminals of ipsilateral and contralateral axons arising from the same part of the retina at such an early stage of development implies very strongly that these populations of axons respond differentially to guidance cues in the SC. In order to confirm that these results were representative of retinal projections in general, we also performed injections into nasal retina. As for ventrotemporal retina, a relatively well circumscribed contralateral TZ was clearly apparent, although, as expected, this was located in caudal, rather than rostral SC (Fig. 2H). Little evidence of branching was observed outside the TZ region.

In animals analysed at P7, the contralateral TZ was much denser than at P4 and better circumscribed, with only 1 or 2 axons continuing caudally past the densely labelled region at the rostromedial border of the SC (Fig. 2I). The ipsilateral TZ was also more obvious than at previous ages, although it remained less dense than the contralateral projection (Fig. 2I). A fairly high proportion of the axons which appeared to innervate the TZ continued caudally for some distance, but did not appear to branch outside the TZ. In contrast to younger ages, the ipsilateral projection had a blurred appearance in whole-mount preparations, presumably relating to the overall growth and maturation of the SC (see Discussion). Horizontal sections through the SC allowed these labelled fibres to be viewed with much greater clarity (Fig. 2I, inset). The caudolateral offset of the ipsilateral projection compared to the contralateral projection was readily apparent, consistent with that seen at maturity.

By P11, the contralateral TZ was similar in density, location and size to the mature TZ (Fig. 2J). The ipsilateral TZ was also much denser compared to younger animals, with fewer axons seen continuing caudally. These caudally projecting axons had few or no branches outside the main TZ. Occasionally axons were seen travelling lateral to the TZ, but these typically did not branch. As at P7, the appearance of the ipsilateral TZ was blurred in wholemounts, but was clearly visible in horizontal sections as a region of increased branch density (inset in Fig. 2J).

By P18 both ipsilateral (Fig. 2K, inset) and contralateral TZs were tightly clustered, and closely resembled the appearance of the mature TZ (Fig. 2K). In some cases, unbranched axons which continued caudally past the TZ were observed (not shown).

The highly stereotyped location of the ipsilateral and contralateral TZs across all developmental stages following our injections into VT retina is clearly visible in figure 2. While the contralateral TZ was always located at the rostromedial corner of the SC, the ipsilateral TZ was always offset to a similar degree. The difference in the location of the ipsilateral and contralateral TZs with respect to the RC and ML SC axes was quantified at P11 and found to be significantly different for both axes (see below). This organisation therefore emerges prior to eye-opening which occurs on P12–13.

While the location of the TZ centres showed no change across development, the relative size of the TZs did appear to change. We therefore used this parameter as an index for the maturity of the projection. Material from P1 animals was excluded from this analysis, as the TZ could not be defined. Quantitative analysis confirmed a highly significant interaction between age and TZ size (Fig. 2L; ANOVA, F(4,18) = 298.03. p<0.001). The size of both ipsilateral and contralateral TZs decreased significantly between P4 and P7 (p<0.001; Tamhane's T2), and showed a significant further decline between P7 and P11 (p = 0.017; Tamhane's T2). No significant difference was observed between P11 and any of the older age points for either ipsilateral or contralateral TZs (p>0.98 for both groups; Tamhane's T2) indicating that the projection can be considered to have attained its mature appearance by P11.

### Ten-m3 is expressed in topographically corresponding gradients in the retinocollicular pathway

**Figure 3:**
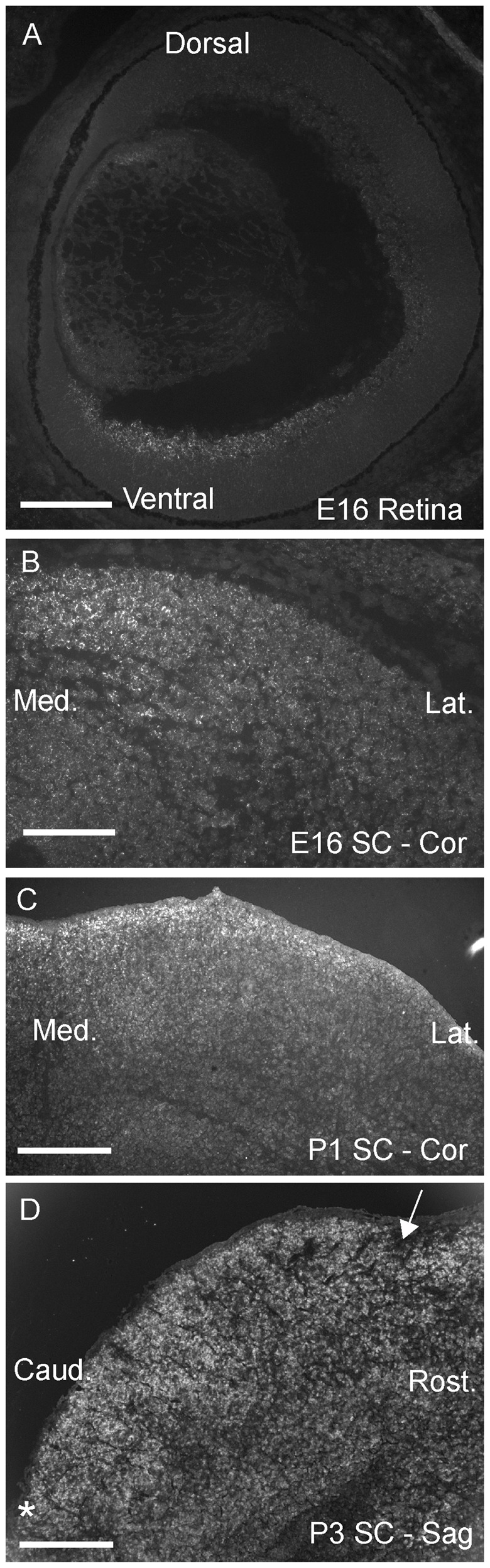
Ten-m3 is expressed in topographically corresponding gradients in the retina and SC. **A:** *In situ* hybridisation for Ten-m3 on a coronal section through the retina of an E16 mouse. Expression shows a high-ventral to low-dorsal distribution in the developing RGC layer. **B–C:** *In situ* hybridisation for Ten-m3 in coronal sections through the SC at E16 (B) and P1 (C). Expression shows a high medial to low lateral distribution in the superficial layers at both ages. **D:** *In situ* hybridisation for Ten-m3 in a sagittal section through the SC at P3. Expression is uniform across the RC axis of the SC. Anterior border of the SC marked with an arrow. Posterior border of SC marked with an asterisk. Scale bars: A: 300 µm; B: 200 µm; C: 250 µm. D: 250 µm. Med: Medial, Lat: Lateral; Rost: Rostral; Caud: Caudal.

To investigate a potential role for Ten-m3 in the formation of the retinocollicular projection we extended our previous analysis of its expression pattern to include both major axes of the retina and SC. An example of the high ventral to low dorsal expression pattern of *Ten-m3* in the retina at E16, revealed by *in situ* hybridisation and consistent with our previous report [23], is provided for reference (Fig. 3A). This pattern of expression is maintained into the first postnatal week [23]. To determine whether a gradient may also be present across the orthogonal, temporonasal, axis of the retina, we used qPCR to determine relative expression levels of *Ten-m3* mRNA in each retinal quadrant. We confirmed the presence of an approximately linear gradient across the ventrodorsal retinal axis (fold change and p values with respect to dorsal retina ( = 1); temporal = 1.74, p = 0.03; nasal = 2.00, p = 0.03; ventral = 3.02, p<0.001; Pairwise fixed random reallocation test [31]). No evidence of a differential distribution of *Ten-m*3 between temporal and nasal retina was found (p>0.05, Pairwise fixed random reallocation test [31]). *In situ* hybridisation also supported the presence of a uniform distribution of *Ten-m3* across the temporonasal retinal axis (not shown).

We also investigated whether *Ten-m3* mRNA is also expressed in a gradient in the SC using *in situ* hybridisation and real-time qPCR. We observed the presence of a clear high-medial to low-lateral gradient of expression predominantly within the superficial, retino-recipient layers by E16 (Fig. 3B). This pattern was maintained throughout the first postnatal week. Figure 3C shows an example at P1. The high-medial to low-lateral gradient was also clearly visible in wholemounts of SC; a RC gradient was not apparent in this material nor in sagittal sections through the SC at P3 (Fig. 3D). Quantitative analysis with real time qPCR supports the presence of a differential distribution of *Ten-m3* across the ML axis of the SC (at P3, *Ten-m3* mRNA expression was 5.0±1.2, mean ± se, fold higher in medial versus lateral SC p = 0.016; Pair-wise fixed reallocation randomisation test, [31]).

### Ten-m3 is required for normal targeting of ipsilateral retinocollicular projections

We first assessed whether deletion of Ten-m3 has an impact on the overall structure or laminar organisation of the SC using Nissl and cytochrome oxidase stained material from adult and developing WT and Ten-m3 KO animals. No changes were observed (not shown). Previous work has established that lamination of retina and primary visual cortex (V1) is also normal in Ten-m3 KOs [23].

**Figure 4:**
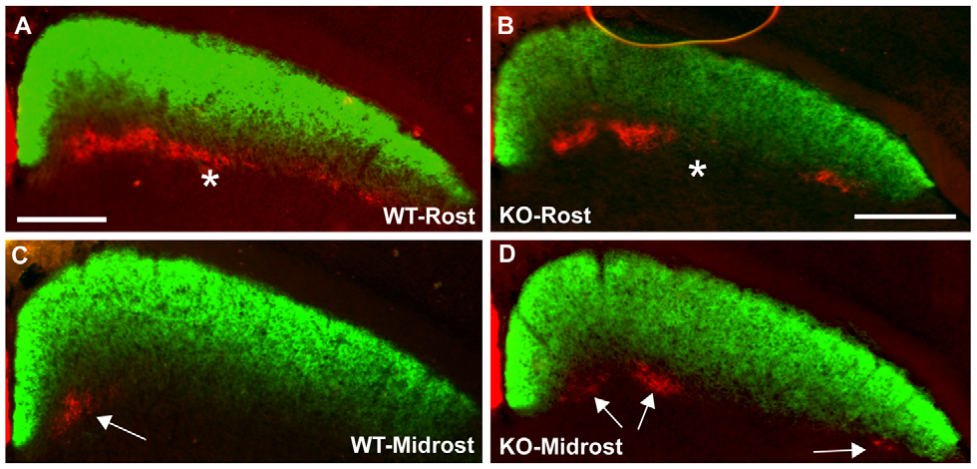
Deletion of Ten-m3 does not disrupt segregation of ipsilateral and contralateral RGC axons or ipsilateral targeting with respect to depth. **A–D:** Coronal sections through rostral (A,B) and midrostral (C,D) levels of the SC in WT (A, C) and Ten-m3 KO (B, D) mice following injections of CTB tagged to red or green fluorescent labels in each eye. Medial is to the left and dorsal to the top in all images. Contralateral terminals (green) form a continuous, dense band in the most superficial layers of the SC in both WT and KO. Ipsilateral axons (red) form clusters in the deep part of the retino-recipient layers of the SC in both WT (A,C) and KO (B,D). Ipsilateral terminals are segregated from contralateral in both WT and KO. In WTs, the ipsilateral axons form a patchy band across the entire mediolateral extent of the nucleus in the rostral SC (A, *) but become quickly confined to one or two patches in medial SC at slightly more caudal levels (arrow in C). In KOs, ipsilateral axons appear slightly less numerous in rostral sections, being absent from some parts of the mediolateral axis of the SC at this level (*). Notably, they do not become confined to medial SC at more caudal levels, but remain scattered across the mediolateral extent of nucleus (arrows in D). Scale bars: 500 µm. Scale in A also applies to C; scale in B also applies to D.

A requirement for Ten-m3 in the targeting of ipsilateral projections was initially assessed using bulk-fill injections of CTB tagged with red or green fluorescent labels to visualise all RGC axons from each eye. In the SC of WT mice, ipsilateral and contralateral retinal axons are segregated with respect to depth, therefore, we first examined coronal sections to determine whether deletion of Ten-m3 alters this organisation. In WTs (Fig. 4A,C) contralateral axons (green) densely filled the superficial layers of the SC with ipsilateral terminals (red) grouped in clusters, deep to the contralateral terminals where they are confined to the ventral portion of the SGS and SO (Fig. 4A,C). This organisation was largely maintained in Ten-m3 KOs (Fig. 4B,D). Occasionally very small numbers of ipsilateral axons were also seen more superficially in KOs, but this was a very small proportion of the overall label, and was also occasionally seen in some WT sections (not shown).

While the segregation of ipsilateral and contralateral terminals, and targeting with respect to SC layers appeared largely normal in Ten-m3 KOs, there was evidence that the tangential distribution of ipsilateral inputs was disrupted. In WTs, ipsilateral terminals were restricted to rostral and medial regions of the SC. In rostral sections, clusters of ipsilateral terminals were distributed along the entire ML extent of the SC, forming a patchy band across this region (Fig. 4A asterisk). They quickly became confined to medial SC at slightly more caudal levels (Fig. 4C, arrow). This organisation was maintained into caudal SC (not shown). In Ten-m3 KO mice, patches of ipsilateral terminals were present in both medial and lateral regions of rostral SC (Fig. 4B). They appeared less numerous in rostral SC than in WTs, however, and rather than forming a patchy band across the entire ML axis as in WTs, they were absent from some regions across the ML extent (Fig. 4B, asterisk). More caudally, they did not become confined to medial SC but were scattered across the ML axis (Fig. 4D, arrows). These misplaced ipsilateral patches varied in absolute location, number and size, making their distribution difficult to evaluate in the coronal plane.

**Figure 5:**
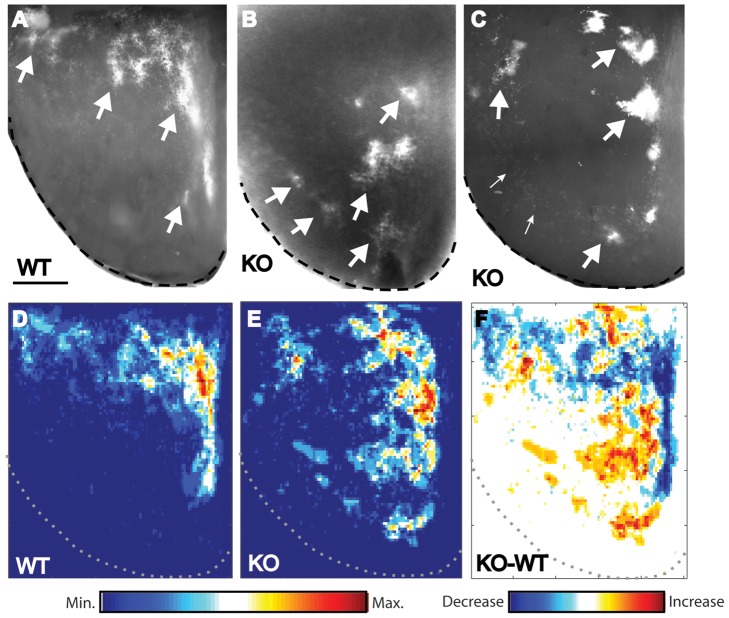
Ten-m3 is required for targeting of ipsilateral axons to rostromedial SC. **A–C:** Horizontal sections through the SC following injection of CTB into the ipsilateral eye from WT (A) and Ten-m3 KO (B, C) mice. Rostral is to the top and medial to the right in all images. In WTs, ipsilateral terminals form clusters that are confined almost entirely to rostromedial portions of the SC (A). Ipsilateral terminals are clustered but are much more widely distributed in Ten-m3 KO mice. In B, terminals largely avoid rostral SC and are seen in mid and even in caudolateral SC. A less extreme case is shown in C. Here, ipsilateral terminals do target rostral SC, but are also seen in more caudal and lateral areas that do not receive ipsilateral innervation in WTs. Some larger clusters of terminals are highlighted by large arrows. Small arrows point to smaller clusters of ipsilateral terminals that were seen in caudolateral SC in some KO cases. **D–F:** Heat-maps indicating overall distribution of ipsilateral label in WT and Ten-m3 KOs compiled across all cases. Rostral is to the top and medial to the right in all images, with dashed lines delineating the borders of the SC. The distribution in WTs is highly stereotyped with label concentrated in rostromedial areas (D). In Ten-m3 KOs ipsilateral terminals are much more widely distributed (E), with clusters seen in more caudal and lateral regions compared to WTs. A subtraction of the WT and KO heat-maps is shown in F to give a clear view of the location and direction of change. Colour bar (D, E): Relative intensity of labelling; Colour bar (F): Difference in intensity between WT and KOs; Scale in A: 500 µm, applies to A–F.

In order to effectively assess the entire distribution of ipsilateral retinal terminals within the SC, horizontal sections through the flattened SC were prepared. The absence of a change in the targeting of the ipsilateral projection with respect to depth means that horizontal sections provide a valid means of comparison. In WTs (*n* = 5), this approach confirmed that ipsilateral projections are grouped in clusters that are almost entirely confined to the rostromedial SC (Fig. 5A). In contrast, the distribution of ipsilateral axons was much more widespread and variable in KOs (*n* = 6). In half of the cases examined, the distribution of ipsilateral axons was profoundly altered with respect to WT, with terminals found almost exclusively in caudolateral regions of the SC (Fig. 5B). In the other half of cases the difference with respect to WT was more subtle, although still apparent: ipsilateral terminals were seen in rostromedial SC, but they appeared less well confined to this region than in WTs, with numerous terminals also seen in more caudal and lateral regions (Fig. 5C).

An expanded distribution of ipsilateral inputs to the SC of Ten-m3 KOs has some similarities with what we have observed in the dLGN [23]. In the dLGN, however, the degree of disruption, as well as the distribution of terminals was much more highly stereotyped between KO animals compared to our current findings in the SC. There was no obvious relationship between the changes in ipsilateral terminal patterning observed in these two structures: all KO animals examined had dramatically altered ipsilateral retinogeniculate projections, consistent with our previous findings, irrespective of the degree of disruption observed in the SC. The more stereotyped effect of Ten-m3 in the retinogeniculate versus the retinocollicular pathway may relate to the increased representation of ipsilateral inputs to the dLGN (15–20% of dLGN area: [11], [32]) versus the SC (2.3%; based on data in [14]), resulting in more stereotyped mapping and/or making changes easier to detect in the thalamus. It may also be indicative of differences in the importance of a given mapping mechanism in the SC versus the dLGN. Other studies have also suggested that connectivity patterns within the SC are differentially reliant on both activity-dependent and specific intrinsic guidance cues compared to the geniculocortical system [33], [34], [35], [36]. The impact of monocular enucleation on the mapping of ipsilateral projections has also been shown to differ between the SC and dLGN [37]. Differences reported in the timing of maturation of retinal TZs in the SC and dLGN may also be reflective of some mechanistic differences between the two structures [38].

To gain an overall measure of the impact of Ten-m3 deletion on targeting of ipsilateral retinocollicular axons, a heat-map compiling all KO and WT data was constructed from normalised, thresholded images. In WTs, the strong bias towards rostromedial SC is clearly apparent (Fig. 5D) with only a few, very small tufts of terminals located outside this region. In Ten-m3 KOs, label is much more widely distributed, with several large, as well as many small patches of terminals in caudal and lateral regions of the SC (Fig. 5E). Subtracted images clearly show the difference between these groups of animals (Fig. 5F), with KOs showing a decrease in label in rostromedial SC (blue colouring) and an increase in caudal and lateral SC (yellow-red colouring).

**Figure 6:**
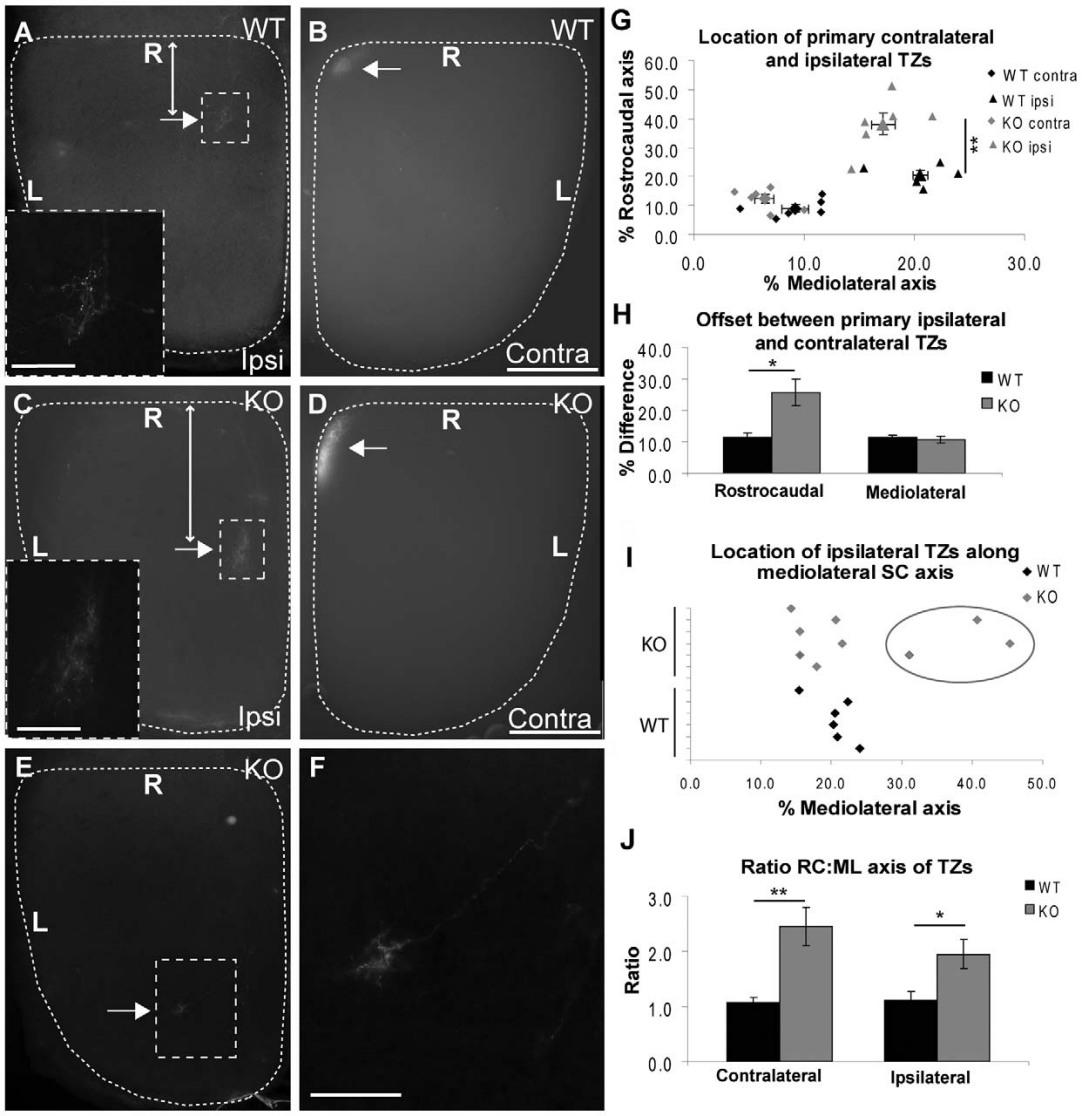
Deletion of Ten-m3 results in mapping changes along the RC and ML axes of the SC. **A–B:** Horizontal section through the ipsilateral SC (A) and whole-mount preparation of the contralateral SC (B) following an injection of DiI into the VTC from a P11 WT mouse. The contralateral TZ forms a densely labelled region near the rostromedial corner of the SC (B, arrow). The ipsilateral TZ (arrow, boxed region also shown at higher power in inset; A) arising from the same injection is offset caudally and laterally, and is located at around 20% of the distance along each collicular axis. The distance of the centre of the ipsilateral TZ from the rostral border of the SC is marked by a double-headed arrow. Borders of the SC are indicated by dashed lines. **C–D:** As for A–B but from a Ten-m3 KO mouse. The contralateral TZ occupies a similar location to that in WT at the rostromedial corner of the SC (D, arrow). The primary ipsilateral TZ occupies a similar location to WT along the ML axis (around 18%), but is shifted much further caudally along the RC axis (41%; C, arrow). Note the increase in the length of the double-headed arrow in the KO (C) which marks the distance of the ipsilateral TZ from the rostral border of the SC compared to the WT example (A). **E–F:** Adjacent (deeper) section to that shown in C, revealing the location of a secondary ipsilateral TZ (boxed region, shown at higher power in F). The secondary TZ is displaced both caudally (at 75% of the RC axis) and mediolaterally (at 40% of the SC axis) from the primary TZ. An additional labelled axon can also be seen (F) but did not form a clear TZ. **G:** Scatter plot showing the location of the centre of the primary contralateral and ipsilateral TZs in 6 WT and 6 Ten-m3 KO mice. Means ± standard errors (s.e.) are also plotted. Contralateral TZs were centred at around 10% of each axis in WT whereas ipsilateral TZs are located significantly further caudally and laterally compared to contralateral TZs, at around 20% of each axis (p<0.05, Student's t-test, see results). The distribution of contralateral TZs in Ten-m3 KO overlaps with that of WT for both axes (p>0.05, Multivariate ANOVA, see results). The location of the primary ipsilateral TZ in Ten-m3 KOs overlaps with that of the WT ipsilateral TZs along the ML axis (p>0.05. Multi-variate ANOVA, see results). The location of the primary ipsilateral TZs are, however, significantly further caudal than in WTs, at around 40% of the RC axis (p<0.01, Multivariate ANOVA, see results). **H:** Graph plotting the offset of the primary ipsilateral and contralateral TZs in WTs and Ten-m3 KOs. Their separation along the ML axis of the SC is identical between KOs and WTs (p>0.6, Student's t-test, see results), but is significantly increased along the RC axis in KOs (p<0.05, Student's t-test, see results). **I:** Plot showing the distribution of both primary and secondary ipsilateral TZs along the ML axis of the SC. The presence of a laterally-shifted, secondary TZ in 3 out of 6 KOs is highlighted by the grey ring. Laterally-shifted, secondary TZs are not observed in any WTs. **J:** Graph illustrating a significant increase in the ratio of RC to ML (RC:ML) axes of TZs in the SC for both contralateral and ipsilateral retinocollicular projections in Ten-m3 KOs (Contra: p<0.01; Ipsi: p<0.05, Multivariate ANOVA, see results for details). Scale bars: B, D: 500 µm; Bar in B also applies to A. Inset in A: 150 µm. Inset in C: 100 µm. Bar in D applies to C and E. Bar in F: 200 µm. Rostral is to the top in all images. R: Rostral; L: Lateral. * p<0.05; ** p<0.01.

In order to gain more information as to the nature of the ipsilateral mapping defect, and to determine how this relates to contralateral topography, we performed focal injections of DiI into peripheral ventrotemporal retina at P10 and examined tissue at P11, when both projections have attained their mature form (see Figs 1 and 2). In WTs we consistently labelled a contralateral TZ abutting the rostromedial corner of the SC (Fig. 6B), with an ipsilateral TZ offset caudolaterally (Fig. 6A) identical to the data presented for P11 C57/Black6 mice in figure 2. Given this similarity between the groups, for quantitative analysis, data from P11 C57/Black6 mice (*n* = 2) was combined with that from WT littermates (*n* = 4) of Ten-m3 KOs. The relationship between the ipsilateral and contralateral TZs was quantified by calculating the relative position of the centre of the TZ along both the RC and ML axes of the SC (Fig. 6G). This revealed that the location of the TZs following our peripheral VT injections was highly consistent and reproducible for both contralateral (RC: 9.0±1.3%; ML: 9.1±1.2%) and ipsilateral (RC: 20.6±1.6; ML: 20.6±0.7; mean ±se, *n* = 6 for all measurements) projections. The position of the contralateral and ipsilateral TZs were significantly different from each other (p<0.001 for ML axis and p = 0.015 for RC axis, Student's t-test following Bonferroni correction for multiple comparisons).

In Ten-m3 KOs, we consistently observed a single, contralateral TZ centred close to the rostromedial corner of the SC (Fig. 6D), similar to the location observed in WTs. The location of contralateral TZs (Fig. 6G; RC: 12.2±1.5%; ML: 6.4±0.9%; mean ± se, *n* = 6) did not differ significantly from values obtained in WTs (multi-factorial ANOVA, between subject comparison of WT versus KO for position of contralateral TZs along the ML (F(1, 10) = 3.307, p = 0.099), and RC (F(1, 10) = 2.625, p = 0.136, axes). Although not significant, a trend was present for both axes suggesting that there may be subtle changes in contralateral TZs in Ten-m3 KOs. This is probably related to differences in the shape of the TZs (see below).

For the ipsilateral projection, both the location and number of ipsilateral TZs showed changes with respect to WT. The primary ipsilateral TZ was defined as the largest TZ; in all cases this was the most rostral of all TZs observed. The primary ipsilateral TZ typically appeared to be positioned further caudally than was seen in WTs (Fig. 6C). Quantification confirmed these observations (Fig. 6G). The mean location of the primary ipsilateral TZ in KOs (RC: 38.1±3.8%; ML: 17.2±1.1%; mean ± se, *n* = 6) was almost twice as far caudal as in WTs. This difference was significant (multi-factorial ANOVA, between subject comparison of WT versus KO for ipsilateral TZ along RC axis (F(1, 10) = 14.350, p = 0.004). No difference in the location of the TZ was found along the ML axis for KO versus WT (multi-factorial ANOVA, between subject comparison of WT versus KO for position of ipsilateral TZ along ML axis (F(1, 10) = 3.043, p = 0.112). The offset of ipsilateral and contralateral TZs within KOs was significant (p<0.001 for RC and ML axes, Student's t-test following Bonferroni correction for multiple comparisons). Tracer injections into dorsal retina revealed no change in contralateral mapping, and did not result in ipsilateral labelling (not shown).

We also analysed the difference in the locations of the primary ipsilateral and contralateral TZs for each animal. The offset between the ipsilateral and contralateral TZs was extremely consistent for WTs (ML: 11.4±0.4%, RC: 11.6±1.2%), confirming the reproducibility of our injection protocol. The primary ipsilateral TZs in KOs were offset significantly further caudally from the corresponding contralateral TZ than in WTs (25.9±4.3%, mean ± se, *n* = 6, p = 0.018, Student's 2-tailed t-test). The mean degree of offset along the ML axis showed no change between WT and KO (10.8±1.1%, mean ± se, *n* = 6, p = 0.628, Student's 2-tailed t-test). These data are presented in figure 6H.

In addition to changes in the position of primary ipsilateral TZs discussed above, in half of the Ten-m3 KO mice (3 out of 6 animals where clear ipsilateral terminals were labelled), we observed the presence of an additional, ipsilateral TZ which was displaced not only caudally (at 50–80% of the SC axis), but also laterally from the primary TZ. In the case shown in figure 6C, D the secondary TZ was located in the adjacent section and is shown in figure 6E, F (this slight shift with respect to depth was not seen in the other cases). The secondary TZs were typically smaller than the primary TZs and appeared to emanate from an axon or axons which passed through, or very close to, the primary TZ, but then continued caudolaterally. The fact that the secondary TZs occurred in only a subset of cases precluded quantitative analysis. A plot showing the distribution of primary and secondary ipsilateral TZs across the ML axis of the SC is given in figure 6I. A secondary contralateral TZ was not observed in any animals. The presence of a secondary, caudolaterally displaced ipsilateral TZ in half of the KOs correlates well with the analysis of CTB injections, where dramatic mapping changes were observed in half of the cases examined and changes were present, though less dramatic in the other half (see Fig. 5). Importantly, these mapping defects occur in the absence of any change in the number or position of ipsilaterally projecting RGCs [23].

Finally, we asked whether, in addition to the dramatic changes in ipsilateral mapping described above, there were any more subtle changes in retinocollicular organisation in Ten-m3 KOs, with regard to the size and/or shape of the primary TZs. Analysis of TZ size as a percentage of SC area revealed no change between WT and KO for either contralateral or ipsilateral TZs (contralateral WT: 1.24±0.33%;contralateral KO: 1.19±0.15%, *n* = 6, multivariate ANOVA, WT versus KO comparison of contralateral TZ area, F(1,10) = 0.020, p = 0.889); ipsilateral WT: 0.70±0.12%; ipsilateral KO: 0.63±0.12%, *n* = 6, multivariate ANOVA WT versus KO comparison of ipsilateral TZ area, F(1,10) = 0.139, p = 0.717). Although no difference in total size was present, we noticed that TZs typically appeared more elongated along the RC axis of the SC in KOs compared to WTs (Fig. 6A–D). While the length or width of the TZ expressed as a percentage of the RC (contralateral WT: 9.8±1.7%, contralateral KO: 14.6±1.4%, mean ± se, *n* = 6, multi-factorial ANOVA F(1,10) = 4.663, p = 0.056; ipsilateral WT: 7.2±0.6%, ipsilateral KO: 9.5±2.3%, mean ± se, *n* = 6, multi-factorial ANOVA F(1,10) = 0.96, p = 0.35) or ML (contralateral WT: 8.6±0.9%, contralateral KO: 6.7±1.4%, mean ± se, *n* = 6, multi-factorial ANOVA F(1,10) = 1.228, p = 0.283; ipsilateral WT: 6.7±0.7%, ipsilateral KO: 4.5±0.8%, mean ± se, *n* = 6, multi-factorial ANOVA F(1,10) = 4.878, p = 0.052) axes did not reach statistical significance between WTs and KOs, the ratio of the RC and ML axes of the TZs (see Methods for definition) revealed a marked difference between the groups (Fig. 6J). In WTs, this ratio was close to 1 for both ipsilateral (1.1±0.1) and contralateral (1.1±0.1) TZs. In KOs, this ratio was significantly increased, approaching 2 for ipsilateral projections (1.9±0.3, mean ± se, *n* = 6, multi-factorial ANOVA F(1,10) = 7.700, p = 0.02) and >2 for contralateral TZs (2.4±0.3, mean ± se, *n* = 6, multi-factorial ANOVA F(1,10) = 14.2, p = 0.004). Therefore, in addition to the marked changes in the location of ipsilateral TZs, there are also more subtle changes in TZ shape which affect both contralateral and ipsilateral projections.

## Discussion

The visuotopic alignment of inputs which arise from different retinal locations, but view the same point in visual space, is fundamental to binocular vision. This study demonstrates that the template for this organisation is present in the retinocollicular system from early in development. TZs form in topographically appropriate locations by P4, with ipsilateral TZs always offset in a visuotopically appropriate manner from contralateral TZs. This implies that ipsilateral and contralateral axons from the same retinotopic location are differentially responsive to guidance cues in their target. Ten-m3 is normally expressed in a high-medial to low-lateral gradient in the developing SC. In its absence, ipsilateral axons are distributed broadly across the SC, rather than being confined to rostromedial regions as in WTs. Contralateral TZs from the ventrotemporal retina do not show significant changes in location but exhibit subtle differences in shape. The mapping defects seen in the ipsilateral population occur along both the RC and ML collicular axes. Our data demonstrate that Ten-m3 is essential for the formation of visuotopically-aligned retinocollicular projections.

### Normal development of visuotopy in the retinocollicular pathway

Data for the development of contralateral retinocollicular topography are largely consistent with previous studies in mice [2], [11], [38], [39], although a higher degree of order was apparent by P4 than was reported at this age in rats [39]. Axons originating from discrete retinal regions initially project diffusely over much of the SC. Order arises via interstitial branching in the vicinity of the topographically correct TZ by P4, becoming denser and more refined to take on mature characteristics by P11.

The development of topography in the ipsilateral retinocollicular projection has not been previously described in rodents using focal tracing techniques - these are advantageous as they allow direct comparison of the locations of ipsilateral and contralateral TZs which arise from the same retinal site. We found that the generation of ipsilateral topography occurred in parallel with that of the contralateral projection, sharing the same time-course and mode of development, with TZs beginning to form by P4 and exhibiting a mature appearance by P11. Although not quantified here, ipsilateral TZs were notably reduced in density compared to contralateral TZs in all cases and at all developmental stages examined. While in our material this may reflect the fact that only around 15% of VTC cells project ipsilaterally [1], and so the TZ seen following our injections would be expected to be less dense, a recent study using molecular techniques to label single axons indicates that individual ipsilateral retinocollicular TZs also have a reduced density compared to their contralateral counterparts [38]. These authors also noted differences in the shape of contralateral and ipsilateral TZs in the SC. This was not apparent in our data, and may relate to the different techniques used in the two studies. Importantly here, we found that the ipsilateral and contralateral TZs arising from the same retinal location were spatially distinct from the outset. Ipsilateral axons consistently formed TZs at locations that were offset both caudally and laterally, in a visuotopically appropriate manner, from their contralateral counterparts. Thus ipsilateral and contralateral axons arising from the same region of the retina appear to respond differentially to cues present in the target. This implies that the repertoire of cues which mediate guidance in target structures are distinct between these populations, consistent with the presence of eye-specific guidance cues. Such cues have been demonstrated for guidance at the optic chiasm [40], [41], but have not been previously described for ipsilateral axons in the SC.

The terminal locations were also distinct with respect to depth, with ipsilateral TZs located deep to contralateral TZs. This implies that ipsilateral and contralateral axons are also differentially responsive to cues which determine targeting across the dorsoventral extent of the SC; these cues are yet to be discovered. An interesting possibility is that the differences in targeting with respect to depth could also contribute to the differential targeting of ipsilateral and contralateral axons across the tangential plane. Guidance cues could be differentially expressed with regards to distance below the pial surface and thus preferentially influence ipsilateral versus contralateral axons. To our knowledge this has not been reported, however, for any retinocollicular axon guidance cues to date. We saw no evidence of differential expression of Ten-m3 with respect to depth which could explain its preferential impact on ipsilateral TZs.

In our data, ipsilateral axons and TZs could be clearly visualised from the surface until P4: they became blurred by P7 and were largely obscured by P11. This is probably related to the maturation of the SC, particularly with regard to the thickness and optical density of the most superficial layers of the nucleus, rather than altered targeting *per se*. Although the use of horizontal sections, which optimised information on topography, did not allow us to address this issue directly, the data from previous studies indicates that ipsilateral axons both target and form clusters within deep SGS and SO by P4 [11]. This contrasts with the more diffuse projections that are present more superficially and at younger ages [11]. The appearance of ipsilateral TZs at P4 in our focal tracing data, almost certainly correlate with formation of these clusters seen following bulk-fill injections. By P9 the ipsilateral TZs are located in the same layer but, due to the maturation and growth of the SC, this is now located approximately twice as far from the pial surface [12]. Increases in the density of the neuropil with maturation, as well as myelination which commences around P7–10 in the mouse optic nerve [42], are also likely to contribute to the increasing difficulty associated with visualising ipsilateral projections in wholemounts with age.

### Ten-m3 regulates ipsilateral mapping in the colliculus

Ten-m3 plays a key role in ipsilateral retinal mapping to the dLGN [23] but the complex, three-dimensional topography of this thalamic nucleus made it unclear whether the patterning was altered along the representation of the temporonasal or dorsoventral retinal axes. Our current data demonstrate that Ten-m3 also regulates retinocollicular mapping. The advantageous model provided by the SC has enabled us to determine that mapping errors of ipsilateral projections in KOs occur along both the ML and RC collicular axes, suggesting that Ten-m3 may regulate multiple aspects of ipsilateral retinocollicular mapping. The ML mapping changes, which correlate well with Ten-m3's expression pattern, were seen in half of the cases. Surprisingly, however, changes were more prominent along the RC axis of the SC, affecting all cases examined. Contralateral axons map largely normally along both axes, suggesting that most of the mechanisms underlying their guidance remain functional. Further, analysis of retinocollicular TZs has also permitted the detection of subtle changes in their shape which were not apparent in the dLGN. The current data point to potential mechanisms of action for Ten-m3.

#### Ten-m3 impacts mapping across the ML axis of the SC

Ten-m3 is expressed in a high-medial to low-lateral gradient in the SC which corresponds topographically to its gradients in the retina, dLGN and V1 [19], [20], [23]. Further, it is expressed on growing axons and cell bodies and promotes homophilic interactions between cells that express the protein [19]. These features are compatible with a direct role in specifying connectivity, consistent with the chemoaffinity hypothesis proposed by Roger Sperry [43]. Indeed, a direct role for Teneurins in synaptic targeting has been recently demonstrated in *Drosophila* [26], [27]. In these studies Teneurins are reported to undergo homophilic interactions across synapses to match afferents and targets which express similar levels of the protein. If Ten-m3 acts directly as a homophilic targeting molecule in the mammalian visual system, one would predict that deletion of Ten-m3 would result in a lateral shift in the mapping of axons from ventral retina. In support of this possibility, lateral mapping defects were observed to varying degrees in the SC of Ten-m3 KOs.

While our data are consistent with the possibility that Ten-m3 may act directly as a homophilic attractant molecule, the possibility that Ten-m3 may also impact ML collicular mapping indirectly, potentially via regulating the expression of other axon guidance molecules (see below), must also be considered. Mapping of contralateral axons along the ML collicular axis is thought to be determined by the balance of attractive and repulsive forces exerted by high-medial to low-lateral gradients of ephrinB and Wnt3 within the SC, interacting with high-ventral to low-dorsal retinal gradients of EphB and Ryk, respectively [2], [9]. Ten-m3 is expressed in a similar gradient to these molecules in both retina and SC. The presence of an additional, laterally-shifted TZ in 50% of Ten-m3 KOs is similar to the incidence and direction of change that has been reported for the contralateral projection following deletion of EphB2,3 [2] or increased ephrinB expression in the tectum [7]. Secondary TZs also showed a caudal shift, again reminiscent of EphB mutants [2], [44]. These similarities suggest that Ten-m3 could contribute to the same signalling pathway as EphB-ephrinB to mediate dorsoventral retinal mapping onto the ML axis of the SC. It should be noted, however, that in the case of Ten-m3, the effect is largely specific to the ipsilateral population and, thus far, the impact of EphB deletion has only been reported for the mapping of the contralateral projection. Ipsilateral axons do express EphB1 [41], however, and this molecule has recently been shown to play a critical role in retinocollicular mapping [44]. Since Zic2 is specific to the ipsilateral population [40], can regulate EphB1 expression [45], [46], [47] and interactions between members of the Ten-m and Zic families have been reported ([48]; see also below), it is feasible that Ten-m3 could impact ML mapping via alterations in EphB expression. Alternatively, Ten-m3's actions may be distinct or complementary to those of the EphBs. It would be of interest to determine the effects of altered EphB expression on the ipsilateral pathway, and of combined Ten-m3 and EphB deletions on mapping to see if their effects are additive or overlapping.

BMP also helps to mediate ML mapping via both EphB-dependent and independent pathways [8]. The EphB-independent pathway imposes order in the optic tract. Although not formally assessed, no difference in the distribution of axons entering the SC was observed here, making it unlikely that Ten-m3 interacts with BMP signalling.

#### Ten-m3 regulates RC mapping in the SC

While it is not currently possible to separate the direct and indirect effects of Ten-m3 deletion on ML mapping, the marked alterations in RC collicular mapping we observed are somewhat more informative in this regard. Since there is no evidence of a gradient of Ten-m3 along the RC axis of the SC, or the corresponding temporonasal axis of the retina, these observations indicate that deletion of Ten-m3 causes a mapping shift orthogonal to its expression gradient. Indeed, this effect, observed in 100% of cases, was more marked than impact of Ten-m3 deletion on ML mapping suggesting it is a particularly crucial aspect of Ten-m3 signalling in the visual pathway. This is intriguing and makes it unlikely that Ten-m3's sole action in retinal mapping is via direct homophilic interactions. We therefore propose that Ten-m3's role in mapping is more complex, and may be at least in part due to the ability of the intracellular domains of Ten-m proteins to be cleaved and translocate to the nucleus where they can interact with transcription factors [48], [49]. Although not yet shown directly for Ten-m3, sequence analysis suggests that this property is shared by this family member [24], [50]. Ten-m3 may therefore regulate axon guidance via controlling the expression of other guidance cues. Such a mechanism, particularly the regulation of molecules associated with RC mapping, could underlie the requirement for Ten-m3 for the appropriate targeting of ipsilateral RGC terminals along the SC and retinal axes orthogonal to its expression gradient.

The best characterised group of molecules associated with mapping along the RC collicular axis to date are the EphA/ephrinA family. These are reported to undergo repellent interactions to prevent EphA expressing axons arising from temporal and ventrotemporal retina from forming TZs in caudal SC [4], [5], [14], [51]. The caudal shift of ipsilateral TZs from ventrotemporal retina, as well as the elongation of both ipsilateral and contralateral TZs along the RC axis of the SC, therefore, suggests that EphA or ephrinA expression or signalling may be disrupted in Ten-m3 KOs. Consistent with this, the deletion of ephrinA2, 5 results in the caudal expansion of ipsilateral terminals in the SC, strongly suggesting that ipsilateral axons are sensitive to ephrinA gradients [14]. It should be noted, however, that these mutants also exhibit a slight increase in the size of the ipsilateral projection [14], making it unclear to what extent the changes observed in these mutants are due to altered mapping in the target versus inappropriate decussation at the chiasm. The targeting of contralateral axons also shows dramatic aberrations in ephrinA KOs, as revealed by the presence of ectopic TZs [4], [5], [33], [34]. No ectopic contralateral TZs were observed in the current study.

The changes seen here in contralateral axons are unusual – normally positioned but elongated along the RC axis of the SC with no increase in overall TZ area - and may be suggestive of altered expression levels of specific EphAs/ephrinAs. The largely normal mapping of contralateral axons in Ten-m3 KOs makes it extremely unlikely that ephrinA gradients are simply absent: preliminary investigations reveal no overt change in ephrinA expression in the SC (KAG, ND and CAL, *unpublished observations*). Since our data only allowed the evaluation of TZ shape of axons labelled from VT retina, it is unclear whether the changes observed are specific to this population, or will generalise to other retinal regions. Interestingly, deletion of the EphA7 receptor results in an expansion of the region of contralateral temporal retina which projects to rostral SC [6]. Although the different methodologies make it difficult to directly compare this with our findings, this, together with the studies referred to above, suggest EphA7 as a potential candidate for Ten-m3 interaction. Current studies are addressing this possibility. Alternatively, Ten-m3 may be acting via EphA-EphrinA independent mechanisms, as has been suggested for *Phr1* [52]. It is also possible that the changes in TZ shape could be due to direct effects of Ten-m3 on synapse formation.

#### Ten-m3 deletion impacts TZ shape in ipsilateral and contralateral axons

The demonstration that Ten-m3 has an impact on the formation of contralateral retinal projections is consistent with expression data which suggest that Ten-m3 is not confined to ipsilaterally-projecting RGCs, at least postnatally [23]. The effect of Ten-m3 deletion on TZ shape was relatively mild for both ipsilateral and contralateral axons, subtly increasing TZ extent along the RC axis while decreasing it along the ML axis of the SC. These opposing changes only reached significance when the ratio of the two axes was compared. Again, this is supportive of multiple mechanisms of action for Ten-m3 including potential interactions with the molecular pathways associated with mapping along both of these dimensions. Surprisingly, however, while the RC:ML ratio for KO TZs was significantly altered for retinal projections to both sides of the brain, it appeared to have a stronger impact on contralateral than ipsilateral axons, the reverse of its impact on mapping. One possibility is that this is due to technical issues: measurements of the extent of TZs are likely to be more accurate for the dense contralateral projection than for the much sparser ipsilateral projection and this may have impacted the data. Alternatively, it may suggest that the mechanisms which regulate TZ extent and location are partially independent. This is generally consistent with numerous studies which report marked alterations in topography without any obvious or consistent changes in TZ shape [2], [3], [4], [5], [51]. It should be noted, however, that since TZ shape was not formally evaluated in these studies, subtle changes may have been present but overlooked in comparison to the more dramatic mapping effects. Ten-m3's ability to control mapping of ipsilateral axons along both the RC and ML axes of the SC may also contribute to changes in TZ shapes observed in this mutant.

#### There is a differential requirement for Ten-m3 for the mapping of ipsilateral and contralateral projections

The impact of Ten-m3 deletion on mapping is clearly far greater on ipsilateral than on contralateral RGCs. Differences in expression levels of Ten-m3 between ipsilaterally and contralaterally-projecting RGCs are possible and could potentially explain its differential effects on these populations. Ten-m3 expression does not appear to be confined to ipsilaterally projecting RGCs, however, at least at postnatal stages [23]. Interactions of Ten-m3 with other molecules which are predominantly expressed in the ipsilateral population may underlie its differential effects on ipsilateral and contralateral axons. As noted above, Zic2 is a prime candidate here: this transcription factor determines ipsilateral identity via regulation of EphB1 expression [40], [41], [46], [47], and members of the Zic and Ten-m families have been shown to interact with each other [48].

#### Interactions with activity-dependent mechanisms

The demonstration that ipsilateral and contralateral TZs form in distinct locations which are identical to those that will subserve mature visuotopy well before eye-opening rules out the possibility that visually-driven activity is required for their overall alignment. This does not, however, preclude a role for activity in tuning these projections following the onset of vision, which is also reported to occur [12], [13]. Spontaneous retinal waves are thought to be involved in the development of contralateral topography, and may interact with EphA-ephrinA signalling pathways [33], [53]. Disruptions to retinal waves tend to result in appropriately positioned but enlarged contralateral TZs [34], [54]. Since contralateral TZs were normally located, and showed no change in overall size in Ten-m3 KOs, it is unlikely that retinal waves are disrupted in these mutants. Interestingly, a recent study has shown that synchronous activation of the 2 eyes for a few days just before eye-opening can result not only in the partial desegregation of ipsilateral and contralateral retinal inputs, but also in disrupted mapping of a subset of these ipsilateral axons [55]. Disrupted segregation and mapping of ipsilateral retinocollicular axons is also present in mice where serotonin signalling has been disrupted [36]. Further work is required to determine how Ten-m3 might interact with activity-dependent processes in ipsilateral mapping.

### Conclusion

The visuotopic alignment of inputs which arise from different retinal locations but view the same point in visual space is fundamental for binocular vision. The template for visuotopy resides in the differential guidance of ipsilateral and contralateral retinal axons in the SC. Ten-m3 plays a critical role in this process. Through direct and/or indirect control of axon guidance, this glycoprotein is crucial for the targeting of ipsilateral retinal axons along both the ML and RC collicular axes, suggesting a particularly important role in coordinating ipsilateral mapping along both these dimensions. It also plays a minor role in shaping TZs, for contralateral and ipsilateral projections. Ten-m3 thus makes a critical contribution to the accurate representation of the visual world in the midbrain.

## Supporting Information

Figure S1 **Injections into more temporal or ventral regions of the VTC produce the expected topographic shifts for both ipsilateral and contralateral projections.** **A:** Retinal wholemount showing an example of a DiI injection into temporal retina (arrow). The outline of the retina is marked with a dashed line. A fiducial cut was made from the nasal (N) retina to the optic disc (OD). **B–C:** Labelling in the SC following the injection shown in A. The contralateral TZ (C) is located at the mid-rostral border of the SC (arrow). The ipsilateral TZ arising from this injection is offset caudally (box and arrow; B). High power image of the ipsilateral TZ (boxed area) can be seen in the inset (B). **D:** Retinal wholemount showing an example of an injection into the ventral retina (arrow). Conventions are the same as for A. **E–F:** Labelling in the SC following the injection shown in C. The contralateral TZ (F) can be seen at the medial border of the SC (arrow). The ipsilateral TZ arising from this injection is offset caudolaterally (box and arrow; E). Inset (E) shows higher power image of ipsilateral TZ (boxed area). Scale in A: 1 mm, applies to D. Scale in C: 500 µm, applies to B. Scale in F: 500 µm, applies to E. Scales in Insets in B and E: 100 µm. R: Rostral, L: Lateral.
(TIF)
