# Supplementary figures and images for: Ten-m3 Is Required for the Development of Topography in the Ipsilateral Retinocollicular Pathway
Source: PLoS One. 2012 Sep 19;7(9):e43083. doi: 10.1371/journal.pone.0043083 (PMC3446960; doi:10.1371/journal.pone.0043083)

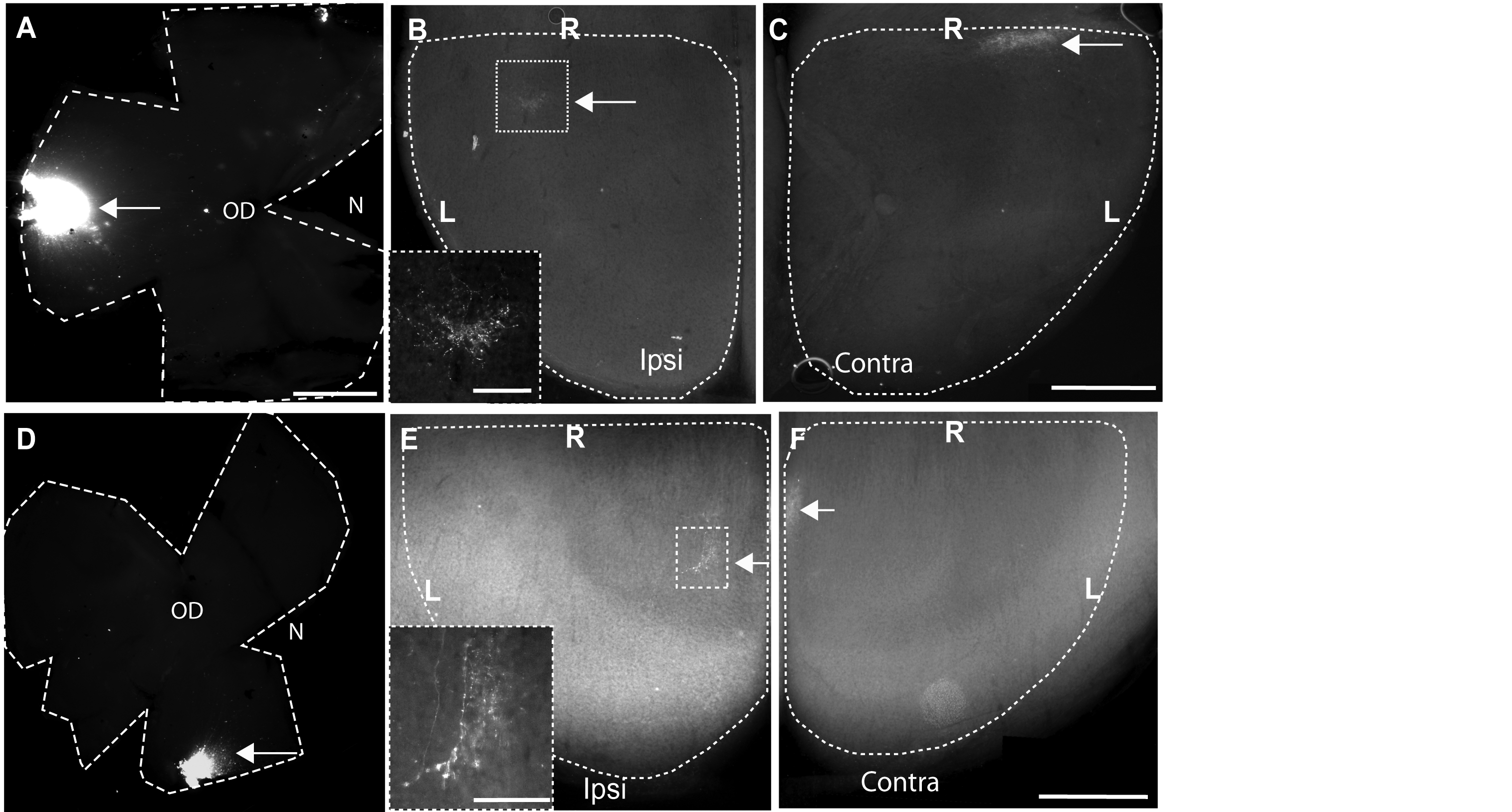

Supplement: Figure S1 — Injections into more temporal or ventral regions of the VTC produce the expected topographic shifts for both ipsilateral and contralateral projections. A: Retinal wholemount showing an example of a DiI injection into temporal retina (arrow). The outline of the retina is marked with a dashed line. A fiducial cut was made from the nasal (N) retina to the optic disc (OD). B–C: Labelling in the SC following the injection shown in A. The contralateral TZ (C) is located at the mid-rostral border of the SC (arrow). The ipsilateral TZ arising from this injection is offset caudally (box and arrow; B). High power image of the ipsilateral TZ (boxed area) can be seen in the inset (B). D: Retinal wholemount showing an example of an injection into the ventral retina (arrow). Conventions are the same as for A. E–F: Labelling in the SC following the injection shown in C. The contralateral TZ (F) can be seen at the medial border of the SC (arrow). The ipsilateral TZ arising from this injection is offset caudolaterally (box and arrow; E). Inset (E) shows higher power image of ipsilateral TZ (boxed area). Scale in A: 1 mm, applies to D. Scale in C: 500 µm, applies to B. Scale in F: 500 µm, applies to E. Scales in Insets in B and E: 100 µm. R: Rostral, L: Lateral. (TIF) [file pone.0043083.s001.tif]
